# Impact of a 3-year mass drug administration pilot project for taeniasis control in Madagascar

**DOI:** 10.1371/journal.pntd.0008653

**Published:** 2020-09-18

**Authors:** Noromanana Sylvia Ramiandrasoa, Pascaline Ravoniarimbinina, Armand Rafalimanantsoa Solofoniaina, Iharilanto Patricia Andrianjafy Rakotomanga, Samuel Hermas Andrianarisoa, Sophie Molia, Anne-Marie Labouche, Anna Sophie Fahrion, Meritxell Donadeu, Bernadette Abela-Ridder, Davidra Rajaonatahina

**Affiliations:** 1 Service de Lutte contre les Maladies Epidémiques et Négligées, Ministère de la Santé Publique Analakely, Antananarivo, Madagascar; 2 Institut Pasteur de Madagascar, Antananarivo, Madagascar; 3 Organisation Mondiale de la Santé Madagascar, Antananarivo, Madagascar; 4 CIRAD, UMR ASTRE, Montpellier, France; 5 Department of the Control of Neglected Tropical Diseases, World Health Organization, Geneva, Switzerland; 6 Faculty of Veterinary and Agricultural Sciences, University of Melbourne, Australia; 7 Initiative for Neglected Animal Diseases (INAND), Midrand, South Africa; 8 Centre Hospitalier Universitaire Joseph Ravoahangy Andrianavalona, Antananarivo, Madagascar; Universidad Nacional Autónoma de México, MEXICO

## Abstract

*Taenia solium* is endemic in Madagascar and presents a significant burden on the population and the health system. The parasite cycles through humans who host the adult tapeworm, and pigs that host the larval stages. Accidental infection of humans may occur with the larval stages which encyst in the nervous central system causing neurocysticercosis, a major cause of seizure disorders and a public health problem. One of the interventions to facilitate the control of the disease is mass drug administration (MDA) of the human population with taeniacide. Here we describe a pilot project conducted in Antanifotsy district of Madagascar from 2015 to 2017 where three annual rounds of MDA (praziquantel, 10mg/Kg) were undertaken in 52 villages. Changes in the prevalence of taeniasis were assessed before, during and after the treatments. A total of 221,308 treatments were given to all eligible people above 5 years of age representing a 95% coverage of the targeted population. No major adverse effects were notified related to the implementation of the MDA. The prevalence of taeniasis was measured using Kato-Katz and copro-antigen techniques. Analyses undertaken combining the results of the Kato-Katz with copro-antigen, or using the Kato-Katz results alone, showed that there was a significant reduction in taeniasis 4 months after the last MDA, but 12 months later (16 months after the last MDA) the taeniasis prevalence had returned to its original levels. Results of the pilot project emphasize the need of a multi-sectorial One-Health approach for the sustained control of *T*. *solium*.

## Author summary

The parasite *Taenia solium* causes neurocysticercosis in humans in its larval stage and is the main cause of seizure disorders in poor communities that keep pigs (the intermediate host). Diagnosis and treatment of neurocysticercosis remains beyond the reach of the general population in Madagascar. Prevention is therefore the most suitable strategy for combating this disease. Mass drug administration (MDA) is used to treat communities for several parasitic diseases such as schistosomiasis. Infection with the *T*. *solium* tapeworm in people can be treated with MDA using praziquantel. Here, we conducted a pilot project in Madagascar, giving over 220,000 treatments to people in 52 villages once per year, for 3 consecutive years. The effects on taeniasis seemed to be encouraging when assessed 4 months after finishing the treatment, but 16 months later, the parasite’s prevalence had returned to its original level. MDA was effective in providing temporary control of taeniasis, but parasite transmission was not interrupted. With respect to *T*. *solium*, this highlights the need of incorporating other interventions such as vaccination and medication in pigs, improved pig husbandry and meat inspection to tackle the whole cycle of the parasite.

## Introduction

*Taenia solium* has been known to be endemic in Madagascar since the early 20^th^ century; porcine cysticercosis was described in 1901, and the first human cases were described in 1904 and confirmed by autopsy in 1909 [1]. Molecular studies of *T*. *solium* from Madagascar found both Asian (Indian subcontinent) and Afro-American genotypes to be widespread on the island, suggesting that the parasite was established in Madagascar at least twice in the past 2000 years [2–4].

The life cycle of *T*. *solium* involves humans and pigs; humans harbour the adult tapeworm (developing taeniasis), and pigs harbour the larval stages (developing porcine cysticercosis). Pigs are infected by ingesting *T*. *solium* eggs released with the faeces of humans harbouring the tapeworm, and humans develop taeniasis after eating undercooked or raw infected pork. The main clinical problems due to *T*. *solium* arise because humans may also be infected with the parasite’s larval stage (cysticercus) by accidentally ingesting *T*. *solium* eggs. In humans, the cysticerci commonly encyst in the brain and the nervous system, causing neurocysticercosis (NCC). NCC is a frequent cause of seizures in countries in which the disease is endemic [5] including Madagascar [6]. NCC is an important problem in many countries where sanitation is poor, and pigs roam freely [7]. The conditions which favour *T*. *solium* transmission and cysticercosis in humans are present in Madagascar, including poor basic sanitation, deficient hygiene practices and free roaming pigs which have opportunities for contact with human faeces.

*T*. *solium* continues to be a health problem in Madagascar [8] and a high endemicity of porcine cysticercosis has been recorded. From March 2013 to February 2014, meat inspection in two abattoirs identified 3,219 pigs out of 68,432 as having cysticercosis, an overall prevalence of 4.7%, adjusted to 21.03% when considering the sensitivity of the meat inspection [9]. Numerous cases of taeniasis were identified in Madagascar when Kato Katz tests on human faecal samples were conducted for epidemiological studies of schistosomiasis and soil transmitted helminths by the Ministry of Public Health in collaboration with the Pasteur Institute during 2012–2016 (personal communication—Sylvia Ramiandrasoa). Of 114 districts sampled, 53 were identified as having cases of human taeniasis. Pork is a major source of meat in most regions of Madagascar, whereas beef is rarely consumed. Similarly, cultural practices in Madagascar do not include consumption of pig raw liver. For these reasons, the majority of the cases of taeniasis identified in the country were believed to be *T*. *solium* and not *Taenia saginata* (that includes cattle as intermediate host) or *Taenia asiatica* (that usually develops in the liver of pigs). Cases of NCC continue to occur, and seropositivity in humans for *T*. *solium* remains high. A serological survey of human cysticercosis including 4,375 samples between 1994–1999, using an ELISA test for screening and EITB (Enzyme-linked Immunoelectro Transfer Blot) for confirmation, showed a prevalence varying from 7 to 21% in the different provinces [10]. In 2013, a cysticercosis survey at the Antsirabe Hospital (Vakinankaratra region) using a commercial Western Blot (LD Bio Diagnostic, Lyon, France) revealed 14.7% seropositivity (35 positives out of 237) [11].

A diverse range of potential control tools are available for *T*. *solium*, and can be aimed at humans and/or pigs. They include preventive chemotherapy for taeniasis, health promotion and education, improvement of basic sanitation, pig vaccination and treatment, meat inspection and improvement of pig rearing conditions [12, 13]. There is not a universal and validated strategy for *T*. *solium* control that can be applicable to all countries or endemic areas. The tools used and the frequency of their usage depend on the resources and capabilities locally available.

A national policy for the control of *T*. *solium* was launched in Madagascar in 2005 with the objective of reducing the impact of cysticercosis. In 2011, preventive chemotherapy implemented as mass drug administration (MDA) was conducted in three districts involving approximately 240,000 people, but it was not possible to conduct a detailed evaluation of the program’s effectiveness.

In presenting a rationale for investment and action for *T*. *solium* control in 2016, the World Health Organization (WHO) emphasised that tools and approaches are available to facilitate the control of *T*. *solium* in humans and animals and used Madagascar as one of the country case examples identifying lessons, context and funding requirements [14].

The government and the Ministry of Public Health of Madagascar remain committed to control the disease and to implement integrated approaches on the fight against neglected tropical diseases. As part of that commitment, here we describe the evaluation of a pilot project for the mass treatment of taeniasis conducted in Antanifotsy district of Madagascar from 2015–2018 with support from the WHO.

## Materials and methods

**Fig 1:**
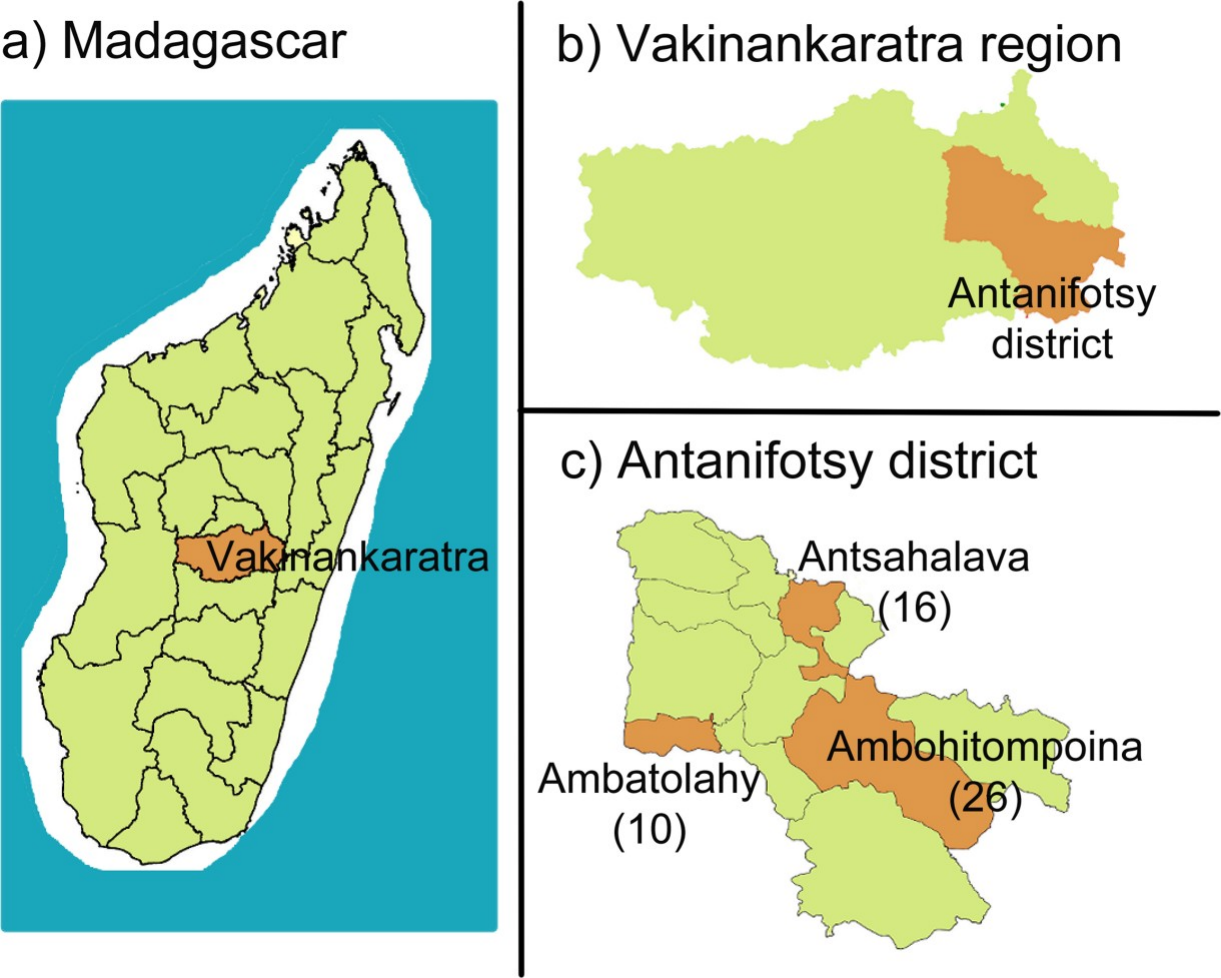
Map of the intervention area. a) Map of Madagascar showing Vakinankaratra region; b) Map of Vakinankaratra region showing Antanifotsy district; c) The three communes targeted (and the number of villages targeted in brackets): Ambatolahy (10), Ambohitompoina (26) and Antsahalava (16).

Madagascar is an island country in the Indian Ocean, off the coast of East Africa. It has 22 regions, which are further subdivided into 116 districts. The district of Antanifotsy (Fig 1) located in the Vakinankaratra region, was selected for the pilot project due to its high prevalence of taeniasis identified during helminthiasis/schistosomiasis surveys conducted by the Ministry of Public Health and the Pasteur Institute. In 2011 the prevalence of taeniasis in villages of Antanifotsy ranged from 2.7 to 19.2%. This area is not endemic for schistosomiasis and there has been no MDA conducted for schistosomiasis control. The district of Antanifotsy comprises 12 communes and 248 fokontany (villages). In 2015 the total population of Antanifotsy was 401,706 with a density of 117 inhabitants/Km^2^. It is an agro-pastoralist area. Three communes, in which the schools showed a higher prevalence of taeniasis during the schistosomiasis mapping, were targeted for the pilot trial (Ambatolahy, Ambohitompoina and Antsahalava) with a total of 52 villages representing 94,862 inhabitants.

The objective of the pilot project was to reduce the prevalence of taeniasis to below 1% in the three communes at least one year after three rounds of annual treatment (2015–2017) of the population by MDA.

### Ethics statement

Ethical authorisation was provided by the Ministry of Public Health of Madagascar (Ref.036/MSANP/CE du 06/06/2015). Informed written consent was obtained from the head of the households and measures were taken to ensure patient confidentiality.

### Mass drug administration

The taenicide drug chosen was praziquantel [15, 16], which is available in Madagascar as it is already used in other regions for control of schistosomiasis. Praziquantel was given as a single dose at 10 mg/kg, which is the WHO recommended dose for taeniasis [15] and is lower than the dose used for MDA for schistosomiasis (40 mg/kg). Adults and children over 5 years of age were included in the MDA project. Community Health Agents (CHA) provided the treatment (there are usually two CHA per village), and Health Agents (HA) of the Ministry of Public Health acted as supervisors. Pregnant women and people with symptoms which might occur in neurocysticercosis (a history of intense or chronic headache, seizures or epilepsy) were excluded due to the potential of praziquantel to cross the blood-brain barrier and precipitate adverse neurological effects due to an inflammatory response of the damaged cysts [17]. The CHA are volunteers who are committed to work for the health of their communities. They receive training in several areas of health and health programs. They are familiar with the community and likely to be aware of people in the area with epilepsy. They received specific training for identification of epilepsy cases prior to commencement of each MDA campaign.

The main activities conducted for the MDA were training of the CHA and HA, social mobilisation (advocacy, materials developed for information, education and communication, awareness campaigns, media coverage, official launch ceremony), treatment and supervision.

Adverse effects to the praziquantel medication were monitored actively and passively. During the 5–7 days over which the MDA administration occurred, the CHA would circulate through the villages and hamlets to identify any possible adverse reactions to the MDA. If a CHA was notified of a reaction, it was recorded in the treatment and report cards, with the evolution of the effects. Minor side effects (MSE) were defined as those that were mild and of short duration, for example effects which disappeared spontaneously or after a brief period of rest and having a drink of water. Major side effects included those which were intense, prolonged and needed medication or medical attention. MSE such as slight nausea and vomiting or digestive disorders were handled by the CHA. Any more significant side-effect such as dizziness, severe headache or seizures were to be referred immediately to the Health Centres, to be dealt by medical personnel. The major side effects were to be reported to the Pharmacovigilance Service of the Ministry of Health, and this was the responsibility of the HA, using the pharmacovigilance sheets available at the Health Centres.

### Impact evaluation

Cross-sectional surveys were conducted in the three communes by faecal examinations for the presence of taeniasis in a sample of the population before starting the MDA (T0), after the first MDA (T1) as well as 4 (T2) and 16 months (T3) after the final MDA. Examination of the faecal samples was conducted by microscopy using the Kato-Katz technique and copro-antigen. The protocol used for Kato-Katz was the one described by the WHO [18]. The copro-antigen technique used was as described by Somers et al [19] with reagents and training provided by the Institute of Tropical Medicine, Antwerp, Belgium (ITM). Samples with an optical density above 0.175 were considered positive. The tests were conducted at the Centre Hospitalier Universitaire–Joseph Ravoahangy Andrianavalona (CHU-JRA) with the support of ITM.

A sample size of 960 was calculated for the faecal examinations using StatCalc from Epi Info™, taking into account that the population over 5 years of age in the 3 communes was 75,668 inhabitants, a taeniasis prevalence of 5.1% based on the results of a survey undertaken in the area in 2011, a 95% confidence interval, an accepted error of 3%, a design effect of 4 and an anticipated non-response level of 15%. Twenty villages from the three communes were selected at random and 48 individuals were selected at random at each testing point for faecal sampling in each village.

Differences in the prevalence of taeniasis were evaluated using the N-1 Chi-squared test using WINPEPI [20, 21].

### Timelines

**Fig 2:**
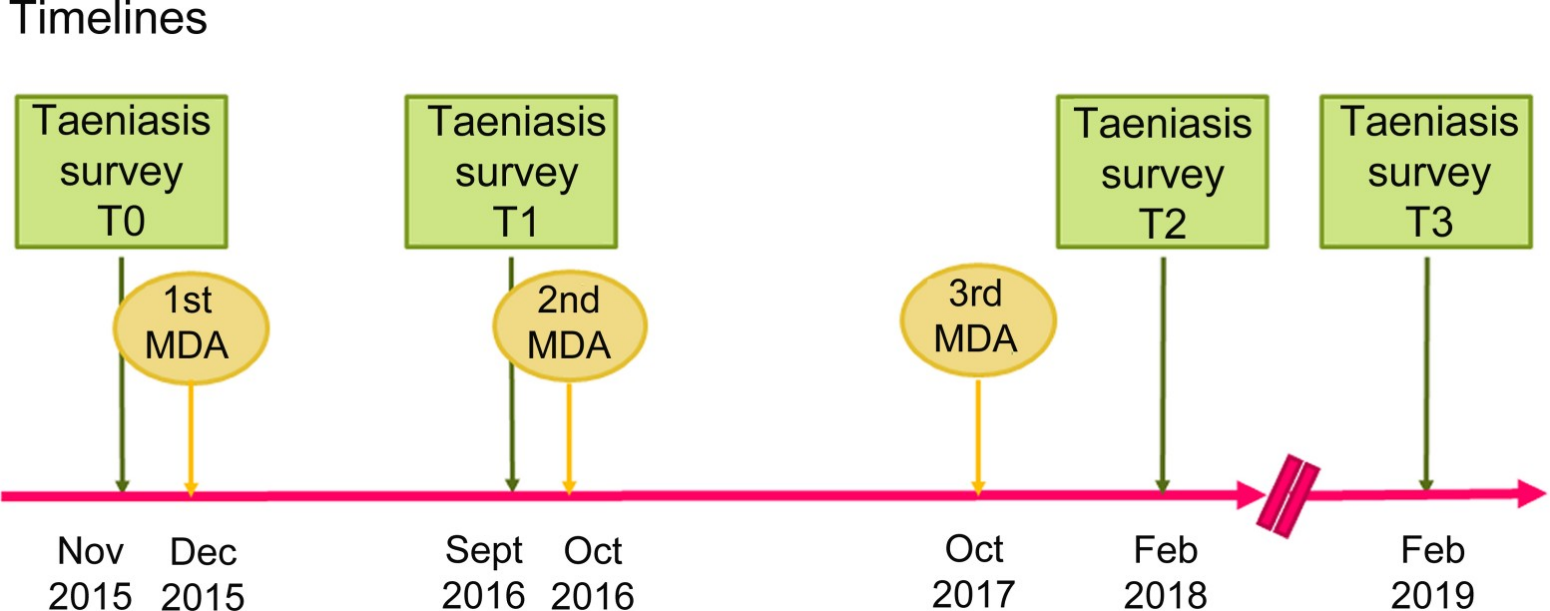
Timelines of the 3-year MDA pilot project for taeniasis control in Madagascar.

The three rounds of MDA were conducted in December 2015, October 2016 and October 2017 (Fig 2). Monitoring surveys were undertaken before the start of the project (Baseline T0: Nov 2015), after the first MDA (Interim evaluation T1: Sept 2016), after the 3^rd^ MDA (T2: February 2018) and a year later, in February 2019 (T3). Originally T3 was planned to be conducted a year after the MDA (October 2018), but it was delayed due to a plague outbreak.

## Results

A total of 208 CHA (the individuals who provided the treatment) and 13 HA (supervisors) were trained. Advocacy material was developed and distributed including awareness posters, campaign launch posters, flyers, banners, etc. Advocacy meetings were held with key stakeholders in 2015, 2016 and 2017. They were all well attended, and the participant showed a high level of interest.

A total of 221,308 treatments were given during the 3 MDA campaigns (Table 1). The population coverage over the 3 years was 95.64%. The coverage rate was calculated as the proportion of the number of targeted people (>5 years of age) who received treatment in each round.

**Table 1:** Coverage of the 3-year MDA pilot project for taeniasis control in Madagascar.

Communes	1^st^ MDA—2015			2^nd^ MDA—2016			3^rd^ MDA—2017			TOTAL		
	P	T	C (%)	P	T	C (%)	P	T	C (%)	P	T	C (%)
Ambatolahy	16,172	15,303	94.63	16,857	16,308	96.74	17,301	17,005	98.29	50,330	48,616	96.59
Ambohitompoina	35,677	34,285	96.10	38,459	36,154	94.01	39,536	37,751	95.49	113,671	108,190	95.18
Antsahalava	21,773	20,284	93.16	22,496	21,546	95.78	23,128	22,672	98.03	67,397	64,502	95.70
Total	73,622	69,872	94.91	77,811	74,008	95.11	79,965	77,428	96.83	231,399	221,308	95.64

P: Total number of people > 5 years of age; T: Number of people treated; C: Coverage

Adverse side effects were monitored by the CHA and HA according to the project protocols. During the three rounds of MDA, minor side effects (MSE) such as nausea and temporary diarrhoea were noted in 0.37%, 0.25% and 0.22% of the persons treated for each round (Tables 2 and 3). The majority of MSE were digestive, and they resolved after drinking water and ingesting some food. There were no notifications of major side effects.

**Table 2:** Minor side effects (MSE) recorded during a 3-year MDA pilot project for taeniasis control in Madagascar using praziquantel 10mg/kg in patients > 5 years of age.

Communes	MSE 1^st^ MDA 2015					MSE 2^nd^ MDA 2016				
	T	D	A	0	Total (%)	T	D	A	0	Total (%)
Ambatolahy	15,303	41	8	14	63 (0.41%)	16,308	36	4	5	45 (0.28%)
Ambohitompoina	34,285	75	12	10	97 (0.28%)	36,154	57	17	8	82 (0.23%)
Antsahalava	20,284	67	8	27	102 (0.5%)	21,546	31	24	1	56 (0.26%)
Total	69,872	183	28	51	262 (0.37%)	74,008	124	45	14	183 (0.25%)

T: Number of people treated; D: Digestive side effects: nausea, vomits, abdominal pain and mild diarrhoea; A: Allergic reactions: urticaria, itching; O: Others such as weakness.

**Table 3:** Minor side effects (MSE) recorded during a 3-year MDA pilot project for taeniasis control in Madagascar using praziquantel 10mg/kg in patients > 5 years of age (continued from Table 2).

Communes	MSE 3^rd^ MDA 2017					MSE—TOTAL				
	T	D	A	0	Total (%)	T	D	A	0	Total (%)
Ambatolahy	17,005	27	12	1	40 (0.24%)	48,616	104	24	20	148 (0.30%)
Ambohitompoina	37,751	48	17	9	74 (0.20%)	108,190	180	46	27	253 (0.23%)
Antsahalava	22,672	41	11	2	54 (0.24%)	64,502	139	43	30	212 (0.33%)
Total	77,428	116	40	12	168 (0.22%)	221,308	423	113	77	613 (0.28%)

T: Number of people treated; D: Digestive side effects: nausea, vomits, abdominal pain and mild diarrhoea; A: Allergic reactions: urticaria, itching; O: Others such as weakness.

Twenty villages were randomly selected for evaluation by examination of faecal samples. Due to logistical issues, only 15 of the original villages selected and included in T0, were accessible at T2 and T3, so an additional 5 randomly selected villages were used to replace those villages which became inaccessible to complete the sample size of 20 villages. During T1, only 5 of the original selected villages were accessible and so for that sampling period another 15 randomly selected villages were included. For this reason, at each point 20 villages were included, with 15 matched between T0, T2 and T3. Results of Kato-Katz and copro-antigen testing are summarised in Tables 4 and 5.

**Table 4:** Results of faecal testing for taeniasis during a pilot control program for *T*. *solium* in Madagascar. Results of Kato-Katz and copro-antigen tests on faecal samples before (T0), after the final round of MDA (T2) of the 3-year pilot project, and one year later (T3). Results are shown for the 20 total villages surveyed.

Time	N	KK+CA-	KK+CA+	KK-CA+	Total KK or CA pos	% KK or CA Pos		Total KK pos only	% KK pos
T0	960	12	7	28	47	4.90%		19	1.98%
T2	976	1	1	4	6*	0.61%		2*	0.20%
T3	960	1	9	30	40	4.17%		10	1.04%

KK: Kato-Katz; CA: Copro-antigen; pos: positive

* statistically significant difference in the proportion compared to T0

**Table 5:** Results of faecal testing for taeniasis during a pilot control program for *T*. *solium* in Madagascar. Results of Kato-Katz and copro-antigen tests on faecal samples before (T0), after the final round of MDA (T2) of the 3-year pilot project, and one year later (T3). Results are shown for the 15 villages matched between T0, T2 and T3.

Time	N	KK+CA-	KK+CA+	KK-CA+	Total KK or CA pos	% KK or CA Pos		Total KK pos only	% KK pos
T0	720	8	5	18	31	4.31%		13	1.81%
T2	733	1	1	3	5*	0.68%		2*	0.27%
T3	720	0	7	25	32	4.44%		7	0.97%

KK: Kato-Katz; CA: Copro-antigen; pos: positive

* statistically significant difference in the proportion compared to T0

Comparing the 20 villages, or only the 15 matched villages, the reduction of the prevalence between T0 and T2 was statistically significant (P<0.001). However, between T0 and T3, there was no significant difference (P>0.05).

Statistically significant reductions were observed between T0 and T2 when comparisons were undertaken combining both Kato-Katz or copro-antigen positive samples, or when comparing only the results of the Kato-Katz test. Comparisons between T0 and T3 found no significant difference, either when comparing positive results for either Kato-Katz or copro-antigen test, or Kato-Katz alone.

Twenty villages were also surveyed at T1 using the Kato-Katz and the copro-antigen test. They were randomly selected within the area of the MDA; five of these villages coincided with the villages also surveyed at T0, T2 and T3. The number of samples taken was 961, one sample was Kato-Katz positive but copro-antigen negative, and 10 samples were Kato-Katz negative but copro-antigen positive (Table 6). That is 1.14% of the samples were positive. Among the five villages which were assessed at both T0 and the first post-treatment sampling (T1), there were 14 cases of taeniasis with KK or copro-antigen at T0 while 2 positive cases were recorded at T1.

**Table 6:** Results of all faecal samples which tested positive during the MDA pilot control program for *T*. *solium* in Madagascar. Results shown for the 20 villages tested at each time-point. Different batches of reagents were used for the copro-antigen tests undertaken at each time point.

Time	KK+CA-	KK+CA+	KK-CA+	Total KK+	%KK+, also CA+
T0	12	7	28	19	37%
T1	1	0	10	1	0%
T2	1	1	4	2	50%
T3	1	9	30	10	90%

KK: Kato-Katz; CA: Copro-antigen; +: positive; -: negative

## Discussion and conclusions

Approximately 95% coverage of the population targeted at each round of MDA was achieved thanks to the engagement of the local communities and the successful awareness and social mobilisation campaigns. Three annual praziquantel treatments of the human population in 52 villages in Madagascar resulted in more than 80% reduction in the prevalence of taeniasis in the treated communities when measured approximately 4 months after the third treatment. However, the prevalence of taeniasis returned to a level similar to that seen prior to the first MDA, when measured 16 months after the final MDA round.

Changes in the levels of taeniasis infection that were observed were similar when comparing results from different diagnostic procedures and when including the total 20 villages sampled, or the 15 matched villages between T0 and T2/T3. Recording samples as being positive using either direct (Kato-Katz positive) or indirect (copro-antigen test positive) evidence of the presence of a tapeworm, or when only definitive evidence for the presence of a tapeworm was included (i.e. Kato-Katz positive due to the presence of taeniid eggs in the faeces), led to similar outcomes in terms of changes in taeniasis prevalence (Tables 4 and 5). Also, the results observed when data from all 20 villages that were involved in the trial were included in the analyses, or when comparisons were restricted to only the 15 villages which had matching data at the start and the end of the pilot project, also led to similar outcomes in terms of changes in the prevalence of taeniasis (Tables 4 and 5).

At the time of the initial post-MDA assessment (T1), five villages were assessed that had also been assessed at T0. Among those five villages there were 14 presumptive cases of taeniasis (Kato-Katz or copro-antigen positive) at T0 and only 2 positive cases recorded at T1. One was Kato-Katz positive and the other only positive in the copro-antigen test. The assessment at T1 was undertaken approximately 9 months after treatment. These initial data suggested that the MDA treatment was having a substantial impact on taeniasis in the communities.

Assessment T2 was undertaken approximately four months after the third MDA treatments. The pre-patent period for *T*. *solium* is approximately eight weeks [22, 23]. Positivity in copro-antigen tests occurs at approximately the time of patency [24]. For this reason, the assessment undertaken at T2 gives an indication of the number of new infections acquired only in the period within approximately two months after the last MDA. On this basis, the occurrence of 5 new infections representing 0.68% of the population seen at T3 could be extrapolated, over a 12-month period, to re-establishment of a prevalence of a 4.08% infection over 12 months in the absence of further MDA, suggesting a relatively rapid re-establishment of taeniasis infection. This estimate appears to have been corroborated by the assessment undertaken at T3, 16 months after the last round of MDA, which confirmed that taeniasis levels had returned to a similar prevalence to that seen prior to the start of MDA.

Among the faecal samples tested during the project at all times, more samples were detected as being positive in the copro-antigen test than were detected by Kato-Katz (Table 6). In our studies at T0, of 19 faecal samples that were tested and found to have taeniid eggs present (Kato-Katz positive), only 7 (37%) were positive in the copro-antigen test, suggesting a substantially lower sensitivity for the test than that observed by Mwape et al. [25], but closer to the results recorded from Vietnam where only 1 out of 7 (14%) Kato-Katz positive samples also tested positive with the copro-antigen [26]. The number of Kato-Katz positive at T1 and T2 is too low to analyse. Significantly different results were observed with the copro-antigen test which were conducted at T3 in comparison to the results of T0 (T0; P = 0.008, Fisher’s Exact Test). This change in the test’s performance might be related to a change in the reagents used in the test, which incorporates a polyclonal rabbit anti-*Taenia* antiserum [25], as different batches were used at each testing time. These results emphasize the difficulties in conducting copro-antigen tests for taeniasis as there are no commercial tests available, and the validation and quality control of in-house produced reagents is complex. Of 89 faecal samples that tested positive by the coproantigen test, only 17 (19%) were also positive for the presence of taeniid eggs. This is not unexpected because Kato-Katz is known to have a relatively low sensitivity for detection of taeniasis [26, 27]. Nevertheless, despite its low sensitivity the Kato-Katz test alone was able to detect significant differences in the prevalence of taeniasis given the prevalence of taeniasis in the study population and the sample size.

This study did not differentiate which species of *Taenia* was responsible for causing taeniasis. Three species cause taeniasis in humans–*T*. *solium*, *T*. *saginata* and *T*. *asiatica*. *T*. *saginata* has not been reported in Madagascar and it is unclear whether *Taenia asiatica* occurs in Madagascar. Fan and colleagues [28] found that pigs experimentally infected with eggs of a *Taenia* tapeworm that was described as having originated in Madagascar developed into cysts in the liver. This has been interpreted as providing evidence for the presence of *T*. *asiatica* in Madagascar. However, the provenance of the tapeworm used by Fan and his colleagues is equivocal. By comparison, there are a number of reports of *T*. *solium* from Madagascar, in both humans and animals (pig) [6, 8, 9]. For these reasons, we consider that many of the cases of taeniasis that were detected, were due to *T*. *solium*. Nevertheless, the praziquantel MDA that was implemented in this trial would have been effective against all three species causing human taeniasis.

There were no major side effects reported after treatment with praziquantel at 10mg/kg. This could be because all suspicious cases of neurocysticercosis were excluded from treatment and/or because of the dose of praziquantel that was used. The praziquantel dose commonly used for taeniasis, and that was used here, is 10mg/kg. This is much lower than the dose levels used for the treatment of cysticercosis when it is desired that the drug crosses the blood-brain barrier and affects the parasite (50mg/kg daily in three divided doses for 14–30 days, or 25mg/kg given two-hourly in a single day scheme, or combined with albendazole at 50 mg/kg for 10 days) [15, 29, 30]. A recent review on drugs used for preventive chemotherapy for taeniasis [16], did not find evidence of severe side effects associated to the use of praziquantel at this dose. There has been a limited number of case reports of adverse events in neurocysticercosis patients, but it is unclear whether these reactions were coincidental occurrences. However, due to the limited evidence, it concluded that praziquantel at 10mg/kg can be considered for mass drug administration of taeniasis, but active monitoring of side effects is warranted to minimise concerns and to add to the evidence of its safety. Several authors have evaluated the reduction in taeniasis in human populations following MDA with a taeniacide (reviewed by Lightowlers [12]). Most trials in which MDA alone has been implemented have been undertaken over a small number of years and sustained impacts on *T*. *solium* transmission have not been demonstrated. Mathematical modelling published by Brae et al. [31] found that annual MDA with taeniacide with a coverage of 75% of the human population achieved a 50–60% reduction in the prevalence of *T*. *solium* taeniasis when assessed 12 months after completion of 4 annual rounds of MDA. Our results in Madagascar after three annual rounds of MDA, assessed 16 months after the final MDA, suggest that the impact on taeniasis was less than the modelling may have predicted. More recent modelling [32] of *T*. *solium* transmission using a coverage of 80% of the human population, suggests that annual MDA in humans for as long as 10 years would be predicted to be slow in reducing the prevalence of taeniasis, whereas biannual MDA for 10 years could achieve elimination of *T*. *solium* transmission with a probability of >80%.

In the case of *T*. *solium*, the impact of taeniasis treatments alone as a control measure is hampered by the continued existence of infections in the pig population which serve as a source for re-establishment of taeniasis in humans. Mathematical modelling of *T*. *solium* transmission and its control [31–34], and a logical model of *T*. *solium* control [35], suggest that a combination of interventions in the pig population (vaccination and medication) as well as MDA application of taeniacide in humans would have a more rapid, more substantial and longer lasting impact on *T*. *solium* transmission than the use of taeniacides alone. The treatment of human taeniasis, and the interventions in pigs are described as core “rapid impact” interventions, but other supportive measures such as improved sanitation, community education, pig husbandry and meat inspection can also play an important role [36]. At national level, the control of *T*. *solium* requires not only technical activities, but also a socioeconomic approach and political engagement.

While the use of MDA for taeniasis in the Madagascar communities described here did achieve a short term reduction in the prevalence of taeniasis to ≤1%, re-establishment of taeniasis after the cessation of treatments indicates that other control measures would be needed in order to bring about a sustained reduction in *Taenia* transmission. With respect to *T*. *solium*, control trials are warranted incorporating MDA together with other control measures targeting infections in the pig population towards implementation of a national cysticercosis control program.

## References

[1] Migliani R, Rasolomaharo M, Rajaonarison P, Ravaoalimalala VE, Rabarijaona L, Andriantsimahavandy A. [Cysticercosis in the port of Mahajanga: more frequent than we thought!]. Arch Inst Pasteur Madagascar. 2000;66(1–2):39–42. Epub 2002/12/05. . 12463033

[2] Ramanankandrasana-Randrianarivo BM. Variation intraspécifique de *Tænia solium*: analyse génétique par Random Amplified Polymorphic DNA, relation avec la répartition géographique [PhD]: Universite de Limoges; 2003.

[3] Michelet L, Dauga C. Molecular evidence of host influences on the evolution and spread of human tapeworms. Biol Rev Camb Philos Soc. 2012;87(3):731–41. 10.1111/j.1469-185X.2012.00217.x . 22321512

[4] Yanagida T, Carod JF, Sako Y, Nakao M, Hoberg EP, Ito A. Genetics of the pig tapeworm in Madagascar reveal a history of human dispersal and colonization. PLoS One. 2014;9(10):e109002 Epub 2014/10/21. 10.1371/journal.pone.0109002 25329310 PMC4198324

[5] Garcia HH, Del Brutto OH, Cysticercosis Working Group in P. Neurocysticercosis: updated concepts about an old disease. Lancet Neurol. 2005;4(10):653–61. 10.1016/S1474-4422(05)70194-0 . 16168934

[6] Andriantsimahavandy A, Lesbordes JL, Rasoaharimalala B, Peghini M, Rabarijaona L, Roux J, et al Neurocysticercosis: a major aetiological factor of late-onset epilepsy in Madagascar. Trop Med Int Health. 1997;2(8):741–6. Epub 1997/08/01. 10.1046/j.1365-3156.1997.d01-379.x . 9294543

[7] Donadeu M, Lightowlers MW, Fahrion AS, Kessels J, Abela-Ridder B. *Taenia solium*: WHO endemicity map update. Wkly Epidemiol Rec. 2016;91(49–50):595–9. . 27966846

[8] Rasamoelina-Andriamanivo H, Porphyre V, Jambou R. Control of cysticercosis in Madagascar: beware of the pitfalls. Trends Parasitol. 2013;29(11):538–47. Epub 2013/10/23. 10.1016/j.pt.2013.09.002 . 24145061

[9] Porphyre V, Rasamoelina-Andriamanivo H, Rakotoarimanana A, Rasamoelina O, Bernard C, Jambou R, et al Spatio-temporal prevalence of porcine cysticercosis in Madagascar based on meat inspection. Parasit Vectors. 2015;8:391 Epub 2015/07/25. 10.1186/s13071-015-0975-2 26204952 PMC4513394

[10] Andriantsimahavandy A, Ravaoalimalala VE, Rajaonarison P, Ravoniarimbinina P, Rakotondrazaka M, Raharilaza N, et al [The current epidemiological situation of cysticercosis in Madagascar]. Arch Inst Pasteur Madagascar. 2003;69(1–2):46–51. Epub 2005/02/01. . 15678816

[11] Zafindraibe NJ, Ralalarinivo J, Rakotoniaina AI, Maeder MN, Andrianarivelo MR, Contamin B, et al [Seroprevalence of cysticercosis and associated risk factors in a group of patients examined at the Regional Referral Hospital in Antsirabe]. Pan Afr Med J. 2017;28:260 Epub 2018/06/09. 10.11604/pamj.2017.28.260.10463 29881503 PMC5989193

[12] Lightowlers MW. Control of *Taenia solium* taeniasis/cysticercosis: past practices and new possibilities. Parasitology. 2013;140(13):1566–77. 10.1017/S0031182013001005 . 23947762

[13] WHO/FAO/OIE. WHO/FAO/OIE Guidelines for the surveillance, prevention and control of taeniosis/cysticercosis. Murrell KD, editor. Paris, France2005.

[14] World Health Organization. Preventable epilepsy: *Taenia solium* infection burdens economies, societies and individuals: a rationale for investment and action. Geneva, Switzerland: World Health Organization, 2016.

[15] World Health Organization. WHO model prescribing information: drugs used in parasitic diseases. 2nd ed. Geneva, 1995. 146 p. p.

[16] Haby MM, Sosa Leon LA, Lucianez A, Nicholls RS, Reveiz L, Donadeu M. Systematic review of the effectiveness of selected drugs for preventive chemotherapy for *Taenia solium* taeniasis. PLoS Negl Trop Dis. 2020;14(1):e0007873 Epub 2020/01/17. 10.1371/journal.pntd.0007873 31945055 PMC6964831

[17] Garcia HH, Nash TE, Del Brutto OH. Clinical symptoms, diagnosis, and treatment of neurocysticercosis. Lancet Neurol. 2014;13(12):1202–15. 10.1016/S1474-4422(14)70094-8 . 25453460 PMC6108081

[18] World Health Organization. Bench Aid for the diagnosis of intestinal parasites. France: World Health Organization; 1994.

[19] Somers R, Dorny P, Nguyen VK, Dang TC, Goddeeris B, Craig PS, et al *Taenia solium* taeniasis and cysticercosis in three communities in north Vietnam. Trop Med Int Health. 2006;11(1):65–72. Epub 2006/01/10. 10.1111/j.1365-3156.2005.01537.x . 16398757

[20] Abramson JH. WINPEPI (PEPI-for-Windows): computer programs for epidemiologists. Epidemiol Perspect Innov. 2004;1(1):6 Epub 2004/12/21. 10.1186/1742-5573-1-6 15606913 PMC544871

[21] Campbell I. Chi-squared and Fisher-Irwin tests of two-by-two tables with small sample recommendations. Stat Med. 2007;26(19):3661–75. Epub 2007/02/23. 10.1002/sim.2832 . 17315184

[22] Yoshino K. On the subjective symptoms caused by the parasitism of *Taenia solium* and its development in man. Taiwan Igakkai Zasshi (Journal of the Medical Association of Formosa). 1934;33:183–94.

[23] Ito A, Saito M, Donadeu M, Lightowlers MW. Kozen Yoshino's experimental infections with *Taenia solium* tapeworms: An experiment never to be repeated. Acta Trop. 2020;205:105378 Epub 2020/02/15. 10.1016/j.actatropica.2020.105378 . 32057776

[24] Tembo A, Craig PS. *Taenia saginata* taeniosis: copro-antigen time-course in a voluntary self-infection. J Helminthol. 2015;89(5):612–9. 10.1017/S0022149X14000455 . 24945107

[25] Mwape KE, Phiri IK, Praet N, Muma JB, Zulu G, Van den Bossche P, et al *Taenia solium* Infections in a rural area of Eastern Zambia-a community based study. PLoS Negl Trop Dis. 2012;6(3):e1594 Epub 2012/04/06. 10.1371/journal.pntd.0001594 22479664 PMC3313923

[26] Ng-Nguyen D, Stevenson MA, Dorny P, Gabriel S, Vo TV, Nguyen VT, et al Comparison of a new multiplex real-time PCR with the Kato Katz thick smear and copro-antigen ELISA for the detection and differentiation of *Taenia spp*. in human stools. PLoS Negl Trop Dis. 2017;11(7):e0005743 10.1371/journal.pntd.0005743 28686662 PMC5517074

[27] Praet N, Verweij JJ, Mwape KE, Phiri IK, Muma JB, Zulu G, et al Bayesian modelling to estimate the test characteristics of coprology, coproantigen ELISA and a novel real-time PCR for the diagnosis of taeniasis. Trop Med Int Health. 2013;18(5):608–14. Epub 2013/03/08. 10.1111/tmi.12089 . 23464616

[28] Fan PC, Chung WC, Lin CY, Wu CC. Pig as a favorable animal for *Taenia saginata asiatica* infection. Kaohsiung J Med Sci. 2006;22(1):1–13. 10.1016/S1607-551X(09)70213-X . 16570562 PMC11918131

[29] Winkler AS, Richter H. Landscape analysis: management of neurocysticercosis with an emphasis on low-and middle-income countries. Switzerland: World Health Organization, 2015.

[30] Garcia HH. Neurocysticercosis. Neurol Clin. 2018;36(4):851–64. Epub 2018/10/28. 10.1016/j.ncl.2018.07.003 . 30366559

[31] Braae UC, Devleesschauwer B, Gabriel S, Dorny P, Speybroeck N, Magnussen P, et al CystiSim—An Agent-Based Model for *Taenia solium* Transmission and Control. PLoS Negl Trop Dis. 2016;10(12):e0005184 Epub 2016/12/17. 10.1371/journal.pntd.0005184 27984581 PMC5161321

[32] Braae UC, Lightowlers M, Donadeu M. Can we recommend practical interventions to prevent neurocysticercosis? Trends Parasitol. 2019; 35(8):592–595. Epub 2019/05/28. 10.1016/j.pt.2019.04.012 . 31151880

[33] Gabriel S, Mwape KE, Phiri IK, Devleesschauwer B, Dorny P. *Taenia solium* control in Zambia: The potholed road to success. Parasite Epidemiol Control. 2019;4:e00082 Epub 2019/01/22. 10.1016/j.parepi.2018.e00082 30662967 PMC6324015

[34] Sanchez-Torres NY, Bobadilla JR, Laclette JP, Jose MV. How to eliminate taeniasis/cysticercosis: porcine vaccination and human chemotherapy (Part 2). Theor Biol Med Model. 2019;16(1):4 Epub 2019/02/26. 10.1186/s12976-019-0100-x 30803437 PMC6390339

[35] Lightowlers MW, Donadeu M. Designing a minimal intervention strategy to control *Taenia solium*. Trends Parasitol. 2017;33(6):426–34. Epub 2017/02/27. 10.1016/j.pt.2017.01.011 . 28236521

[36] World Health Organization. Report of the WHO expert consultation on foodborne trematode infections and taeniasis/cysticercosis. Vientiane, Lao People's Democratic Republic 12–16 October 2009. World Health Organization, 2011 Contract No.: WHO/HTM/NTD/PCT/2011.3.

